# Clinical Efficacy of Percutaneous Kyphoplasty Combined with Calcitriol and Calcium in the Treatment of Traumatic Nonosteoporotic Vertebral Compression Fractures

**DOI:** 10.1155/2022/3489160

**Published:** 2022-02-27

**Authors:** Shouqian Dai, Xin Lu, Ningning Dai, Xiu Shi, Peng Yang, Peng Peng, Feng Xu

**Affiliations:** ^1^Department of Emergency Medicine, The First Affiliated Hospital of Soochow University, Suzhou 215006, China; ^2^Trauma Center, The First Affiliated Hospital of Soochow University, Suzhou 215006, China; ^3^Department of Obstetrics and Gynecology, The First Affiliated Hospital of Soochow University, Suzhou 215006, China

## Abstract

**Objective:**

The present study investigated the clinical efficacy of percutaneous kyphoplasty (PKP) combined with calcitriol and calcium in the treatment of traumatic nonosteoporotic vertebral compression fractures (TNVCFs).

**Methods:**

The patients were equally divided into a control group and a treatment group by a random number table. The patients in the control group underwent PKP surgery, and the patients in the treatment group received calcitriol and calcium on the basis of PKP surgery. The visual analog scale (VAS) pain scores, Oswestry Disability Index (ODI) scores, the height of the anterior edge of the vertebral body, Cobb's angle, and the level of the bone mineral density of the two groups of TNVCF patients before surgery were recorded and compared, one and six months after surgery.

**Results:**

Thirty-six inpatients with TNVCFs admitted to the trauma center of the First Affiliated Hospital of Soochow University from January 2019 to January 2020 were recruited. There were no significant differences in the VAS and ODI scores, the height of the anterior edge of the injured vertebral body, Cobb's angle, and bone mineral density between the two groups before surgery (*P* > 0.05). The VAS scores, ODI scores, the height of the anterior edge of the injured vertebral body, and Cobb's angle of the two groups of patients after surgery were significantly better than those before surgery. One and six months after surgery, the VAS and ODI scores, the height of the anterior edge of the injured vertebral body, Cobb's angle, and the bone mineral density of the patients in the treatment group improved significantly compared to those in the control group (*P* < 0.05).

**Conclusions:**

PKP combined with calcitriol and calcium medications could significantly relieve pain, alleviate the loss of compressed vertebral height and kyphosis, and improve the spinal function and the life quality of the TNVCF patients.

## 1. Introduction

Trauma is the leading cause of death in the youngest and most productive individuals and the fourth leading cause of mortality in all age groups [1]. Death, disability, and loss of productivity imposed by trauma have imposed significant economic burden and rehabilitation costs on the society and families. Trauma often damages the musculoskeletal system, including traumatic spinal fractures. As increasingly more such injuries occur every day, traumatic spinal fractures can lead to devastating consequences, including pain, deformity, and paralysis [2–5]. Among the spinal fractures caused by this high-energy impact, there is a special type of traumatic nonosteoporotic vertebral compression fracture (TNVCF), which is different from the osteoporotic vertebral compression fractures (OVCFs) in osteoporotic patients, with the latter generally caused by low-energy impacts. Treatment for patients with vertebral compression fractures usually includes bed rest, open reduction, internal fixation, and minimally invasive percutaneous kyphoplasty (PKP).

Since Garfin et al. [6] first reported the clinical application of PKP in 1998, it has been widely used to correct spinal deformities, relieve pain, and maintain spinal stability and has gradually become the most popular surgical treatment for OVCFs. In addition to surgical treatment, active antiosteoporosis treatment is the basis for OVCF treatment since it can improve the therapeutic effect of PKP and reduce long-term complications. Calcitriol and calcium are the most commonly used clinical antiosteoporosis drugs. Calcium is the basic raw material for bone synthesis, and calcitriol can promote bone formation, increase the absorption of calcium in the intestine, and play a crucial role in OVCF treatment. However, the effectiveness of calcitriol and calcium in TNVCFs remains controversial. There is still no unanimous consensus on the management of TNVCFs with PKP.

This study enrolled 36 TNVCF patients admitted to the trauma center of our hospital and explored the effect of combined calcitriol and calcium on TNVCF patients undergoing PKP. Our research tried to provide evidence for clinicians to choose optimal treatment for TNVCF patients.

## 2. Materials and Methods

### 2.1. Inclusion and Exclusion Criteria

The inclusion criteria were as follows: (1) patients presenting with spinal trauma (an accident or severe fall); (2) X-ray and CT scans indicating vertebral compression fractures, with the MRI showing that the injured vertebrae had T1-weighted low signal and T2-weighted high signal, consistent with a diagnosis of fresh vertebral fracture; (3) normal bone mineral density (BMD) (*t*-value ≥ −1); (4) patients' cooperation with the research and effective communication with the researcher; (5) subjects signing an informed consent form.

The exclusion criteria were as follows: (1) patients with severe heart, liver, lung, and other organ diseases or mental and nervous system conditions; (2) vertebral osteomyelitis, vertebral tuberculosis, pathological fractures, and acute infections; (3) neurological impairment, such as spinal cord injury or cauda equina injury; (4) a history of osteoporosis; (5) patients who could not cooperate, could not be followed up, or could not perform imaging examinations on time.

### 2.2. Patient Population

According to the inclusion and exclusion criteria, 36 TNVCF patients were recruited from those hospitalized in the trauma center of the First Affiliated Hospital of Soochow University from January 2019 to January 2020. Thirty-six patients were divided into the control and treatment groups by a random number table (*n* = 18). Each case only had a single-segment lesion. In the control group, there were 6 males and 12 females, 18–56 years of age (average = 35.8 ± 7.4). All the involved vertebral segments were the thoracic (T) and lumbar (L) vertebrae: two cases of T10, three cases of T11, five cases of T12, four cases of L1, two cases of L2, and two cases of L3. In the treatment group, there were eight males and ten females, 19–59 years of age (average = 36.7 ± 8.3). The involved vertebral segments were as follows: three cases of T10, four cases of T11, five cases of T12, three cases of L1, one case of L2, and two cases of L3. There was no statistically significant difference in general information between the two groups (*P* > 0.05); therefore, the two groups were matched and could be compared. All the patients signed informed consent forms, which were approved by the Medical Ethics Committee of our hospital.

### 2.3. Surgical Procedure in the Control Group

In this study, all the PKP procedures were performed by the same spine surgeon in the trauma center. Patients in the control group were treated with a standard PKP surgical procedure [7] and postoperative management. Under C-arm fluoroscopy, the pedicle approach was used for a bilateral vertebral puncture, and then a puncture trocar was gently tapped into the vertebral body through a bone hammer. When the C-arm fluoroscopy showed that the tip of the puncture needle was located at the inner edge of the pedicle and the posterior edge of the vertebral body, the puncture needle was moved forward about 0.5 cm; then, the inner core of the puncture needle was pulled out, and the K-wire was inserted. A working cannula was inserted to obtain a small amount of bone for pathological examination. Then, two expansion balloons were inserted, expanded sequentially, and withdrawn after satisfactorily reducing the compressed vertebral body. The prepared bone cement was slowly pushed into the working sleeve from both sides. C-arm fluoroscopy confirmed that the bone cement penetrated well, with no leakage. The working casing was removed after the bone cement solidified.

### 2.4. Medications in the Treatment Group

After the PKP procedure, the patients in the treatment group were prescribed calcitriol (Rocaltrol, no. J20150011, Shanghai Roche Pharmaceutical Co., Ltd.), 0.25 *μ*g twice daily, and calcium (Caltrate D, no. H10950029, Wyeth Pharmaceuticals Co., Ltd.), 0.6 g daily for six months.

### 2.5. Clinical and Radiographic Evaluation

**Figure 1:**
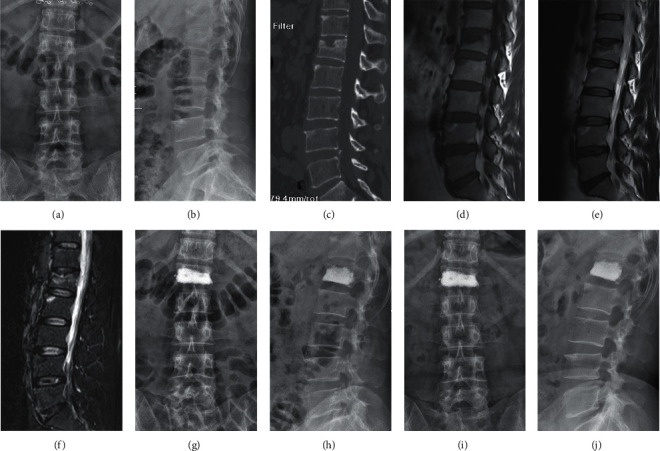
A 44-year-old female patient with TNVCF at L1 in the treatment group. (a) Anteroposterior X-ray film before surgery. (b) Lateral X-ray film before surgery. (c) Vertebral compression fracture shown in the sagittal CT view before surgery. (d) A low-signal intensity in the injured vertebrae shown in the sagittal T1-weighted MRI image before surgery. ((e), (f)) A high-signal intensity in the injured vertebrae shown in the sagittal T2-weighted and short tau inversion recovery (STIR) MRI image before surgery. (g) Anteroposterior X-ray film one month after surgery. (h) Lateral X-ray film one month after surgery. (i) Anteroposterior X-ray film six months after surgery. (j) Lateral X-ray film six months after surgery.

During the entire treatment period, the visual analog scale (VAS) score and Oswestry Disability Index (ODI) score, the height of the anterior edge of injured vertebrae, Cobb's angle, and bone mineral density (BMD) levels of the two groups of patients were recorded before the PKP procedure and one and six months after the procedure. A typical case of a 44-year-old female patient with TNVCFs is shown in Figure 1.

### 2.6. Statistical Analysis

All data conforming to the normal distribution were expressed as means ± standard deviations (*x* ± *s*). Statistical analyses were performed using SPSS 22.0. Comparisons before and after treatment were performed using paired-sample *t*-test, and intergroup comparisons were performed with independent-sample *t*-test. *P* < 0.05 indicated statistical significance.

## 3. Results

### 3.1. VAS and ODI Scores between the Two Groups

Table 1 indicates no significant differences in the preoperative VAS scores between the two groups of TNVCF patients (*P* > 0.05). Compared with the baseline, the VAS scores of the two groups of patients decreased significantly (*P* < 0.05). At 1- and 6-month postoperative intervals, the VAS scores of the patients in the treatment group were significantly lower than those in the control group (*P* < 0.05).

As shown in Table 2, there were no significant differences in the preoperative ODI scores between the two groups (*P* > 0.05). Compared with the baseline, the ODI scores of the two groups of patients decreased significantly (*P* < 0.05). At 1- and 6-month postoperative intervals, the ODI scores of the patients in the treatment group were significantly lower than those in the control group (*P* < 0.05).

### 3.2. Comparison of the Height of the Anterior Edge of Injured Vertebrae and Cobb's Angles between the Two Groups

Table 3 shows no significant difference in the preoperative height of the anterior edge of the injured vertebrae between the two groups (*P* > 0.05). Compared with the baseline, the height of the anterior edge of the injured vertebral body increased significantly in the two groups (*P* < 0.05). At 1- and 6-month postoperative intervals, the height of the anterior edge of the injured vertebrae in the treatment group significantly increased compared with the control group (*P* < 0.05).

Data in Table 4 show no significant difference in the preoperative Cobb's angle between the two groups of TNVCF patients (*P* > 0.05). Compared with the baseline, Cobb's angles in the two groups of patients decreased significantly (*P* < 0.05). At 1- and 6-month postoperative intervals, Cobb's angles of patients in the treatment group decreased significantly compared with the control group (*P* < 0.05).

### 3.3. BMD between the Two Groups

Table 5 shows no significant difference in the preoperative BMD between the two groups (*P* > 0.05). There was no significant difference between preoperative and postoperative BMDs in the control group (*P* > 0.05). In the treatment group, postoperative BMD increased significantly compared with the preoperative BMD (*P* < 0.05). At 1- and 6-month postoperative intervals, the BMDs of the patients in the treatment group increased significantly (*P* < 0.05) compared with the control group.

## 4. Discussion

Traumatic spine fractures most commonly occur in the thoracolumbar vertebrae (especially the T10-L2 region) and can be divided into several types, including compression fractures, stable or unstable burst fractures, flexion-distraction fractures, and fracture dislocations. These patients' need for surgery depends on several factors: the degree of bone compromise, neurological involvement, and the integrity of the posterior ligamentous complex. OVCF is an important factor leading to morbidity and even death of the elderly, and one of its characteristics is low-energy impacts. However, TNVCF, often occurring in young and middle-aged individuals, is often caused by high-energy impacts. TNVCF patients usually experience a high level of pain and disability.

The treatment goals of TNVCFs are to reduce pain, restore vertebral height and mobility, and reduce the risk of vertebral collapse. Treatment options include conservative treatment, PKP, vertebroplasty, open reduction, and internal fixation [8–12]. Conservative treatment includes bed rest, waist protection, functional exercises, and appropriate medications. In the past few decades, minimally invasive PKP has become a popular treatment worldwide for patients with vertebral compression fractures without neurological impairment [13–15]. Many clinical studies have confirmed that PKP fills the balloon in the vertebrae before injecting bone cement to achieve partial reduction [16], relieving pain and stabilizing the fractured vertebral body [17–19]. In this study, all TNVCF patients successfully completed the PKP surgical procedure, and the patients' postoperative VAS and IDO scores were significantly reduced. The heights of the anterior edge of the injured vertebrae and Cobb's angles were restored significantly, indicating that PKP is a safe and effective treatment option for TNVCF patients. Actually, over the last decade, increasingly more surgeons have selected PKP as a viable approach to treat posttraumatic compression fractures [20–22], although PKP was mainly used to treat OVCF initially. The current study confirmed earlier findings. One prospective single-arm study found that percutaneous cement augmentation (PVP and PKP) in TNVCF patients could rapidly relieve pain and significantly improve physical and social functions [23]. In a retrospective study, the authors explored the evolution of vertebral and regional kyphosis in TNVCF patients treated with PKP, reporting that PKP was an efficient and reliable procedure to treat posttraumatic vertebral compression fractures, although there was a slight deterioration of kyphosis correction over time. However, some patients still have a poor prognosis after PKP surgery, accompanied by chronic pain or gradual vertebral collapse. This may be related to the lack of active drug treatment after surgery in some TNVCF patients, which is exactly the focus of this study.

Vitamin D and calcium supplementation is essential to prevent and treat osteoporosis and secondary OVCF. Vitamin D helps absorb calcium and phosphorus from the intestines and maintain bone mineralization and muscle mass and has potential benefits for other organs and systems [24]. Vitamin D can be synthesized in the skin after exposure to the sun and can also be taken in through a balanced diet. With changes in modern lifestyles (reduced outdoor activities and unbalanced diets), in addition to the elderly, some young and middle-aged individuals may also suffer from vitamin D deficiency. Vitamin D deficiency causes osteopenia and osteoporosis in men and women, resulting in bone mineralization defects and muscle weakness, which increases the risk of fractures and refractures after surgery. This is especially true in patients with hip fractures and spine VCFs [25]. Therefore, for the elderly, middle-aged, and young people, especially patients with OVCF or TNVCF, adequate vitamin D levels are a requisite for the efficacy of surgical treatment and comprehensive drug treatment. In addition, it is also important to ensure adequate calcium intake through a balanced diet. Calcium and vitamin D supplements can reduce secondary hyperparathyroidism in the elderly, decreasing the risk of proximal femoral fractures [26].

BMD is a method of measuring bone mass and mineralization by dual-energy X-ray absorptiometry. Past meta-analyses have shown that oral vitamin D3 and calcium supplements in postmenopausal women can increase BMD in the spine, body, femoral neck, and total hip, while oral vitamin D3 supplementation alone is not effective [27]. This sampling test was mainly for menopausal and postmenopausal women, in which the subjects were given 800 IU of vitamin D3 and 500 mg of dietary calcium. In addition, researchers have also found that 400 IU of vitamin D3 and 1000 mg of calcium can significantly increase hip BMD levels [28].

The present study explored the effect of the combined use of calcitriol and calcium on the surgical efficacy and BMD levels in TNVCF patients after PKP surgery. In this study, we administered 0.5 *μ*g of calcitriol (active vitamin D) and 600 mg of calcium per day to the treatment group. The postoperative indicators of both groups were significantly better than the baseline one and six months after surgery. We also found that compared with the control group at the same time intervals, the VAS and ODI scores of the patients in the treatment group decreased significantly after surgery. In addition, the height of the anterior edge of the injured vertebrae and Cobb's angle of the injured vertebrae were restored significantly, and the BMD levels increased significantly. There is growing evidence that vitamin D and calcium are indispensable for bone mineralization, with a key role in fracture healing [29, 30]. A fracture could cause systemic bone loss and reduce 2–15% of total bone mass compared to age-matched controls without fractures [31, 32]. Since secondary posttraumatic bone loss might affect bone mineralization and bone repair and daily dietary vitamin D and calcium supply may not meet the body's requirement for callus mineralization, additional vitamin D and calcium supplements are necessary when a fracture occurs. This partly explains why the postoperative recovery in the treatment group in the present research was significantly better than that in the control group.

However, the present study had some important limitations. First, only small groups of TNVCF patients were included; therefore, a large-scale study of PKP is warranted to reach more convincing conclusions. Second, the duration of the follow-up period was only six months, with possible negative impacts on our findings. Third, we did not determine the levels of vitamin D3 and calcium in blood before and after surgery. Serum calcium concentrations should be better monitored to avoid hypercalcemia.

## 5. Conclusions

PKP can significantly relieve the pain, alleviate the loss of compressed vertebral height and kyphosis, and improve the spinal function and the quality of life of TNVCF patients. The therapeutic effect of PKP combined with calcitriol and calcium medications is significantly better than that of PKP alone, and it is incumbent on surgeons to choose the best strategy to treat patients with TNVCFs.

## Figures and Tables

**Table 1:** Comparisons of VAS scores between the two groups of TNVCF patients before and after the PKP procedure (*x* ± *s*).

Time	Control group (*n* = 18)	Treatment group (*n* = 18)	*t*	*P*
Preoperative	7.98 ± 0.83	7.56 ± 0.67	1.671	0.104
One month after surgery	3.45 ± 0.56^*∗*^	2.99 ± 0.69^*∗*^	2.196	0.035
Six months after surgery	2.66 ± 0.45^*∗*^	2.18 ± 0.56^*∗*^	2.835	0.008

^*∗*^ *p* < 0.05 compared with the preoperative period.

**Table 2:** Comparisons of ODI scores between the two groups of TNVCF patients before and after the PKP procedure (‾*x* ± *s*).

Time	Control group (*n* = 18)	Treatment group (*n* = 18)	*t*	*P*
Preoperative	50.67 ± 7.28	52.34 ± 7.21	0.692	0.494
One month after surgery	34.71 ± 6.08^*∗*^	30.31 ± 5.15^*∗*^	2.343	0.025
Six months after surgery	15.44 ± 3.07^*∗*^	13.02 ± 2.76^*∗*^	2.487	0.018

^*∗*^ *p* < 0.05 compared with the preoperative period.

**Table 3:** Comparisons of the height of the anterior edge of injured vertebrae between the two groups of TNVCF patients before and after the PKP procedure (‾*x* ± *s*, cm).

Time	Control group (*n* = 18)	Treatment group (*n* = 18)	*t*	*P*
Preoperative	1.49 ± 0.11	1.47 ± 0.14	0.477	0.637
One month after surgery	1.96 ± 0.16^*∗*^	2.08 ± 0.19^*∗*^	2.050	0.048
Six months after surgery	1.91 ± 0.15^*∗*^	2.05 ± 0.16^*∗*^	4.256	0.011

^*∗*^ *p* < 0.05 compared with the preoperative period.

**Table 4:** Comparisons of Cobb's angle between the two groups of TNVCF patients before and after the PKP procedure (‾*x* ± *s*, °).

Time	Control group (*n* = 18)	Treatment group (*n* = 18)	*t*	*P*
Preoperative	25.27 ± 3.19	25.09 ± 2.99	0.175	0.862
One month after surgery	17.48 ± 2.21^*∗*^	15.66 ± 2.11^*∗*^	2.527	0.016
Six months after surgery	17.55 ± 2.05^*∗*^	15.83 ± 1.78^*∗*^	2.688	0.011

^*∗*^ *p* < 0.05 compared with the preoperative period.

**Table 5:** Comparisons of BMD between the two groups of TNVCF patients before and after the PKP procedure (‾*x* ± *s*, g/cm^3^).

Time	Control group (*n* = 18)	Treatment group (*n* = 18)	*t*	*P*
Preoperative	0.98 ± 0.09	0.94 ± 0.11	1.194	0.241
One month after surgery	1.03 ± 0.11	1.11 ± 0.12^*∗*^	2.085	0.045
Six months after surgery	1.01 ± 0.14	1.16 ± 0.15^*∗*^	3.102	0.004

^*∗*^ *p* < 0.05 compared with the preoperative period.

## Data Availability

The datasets used during the current study are available from the corresponding author upon reasonable request.
